# A phase 2 study of niraparib concomitant with tumor treating fields in patients with recurrent grade 4 glioma

**DOI:** 10.1093/noajnl/vdag076

**Published:** 2026-04-20

**Authors:** Elizabeth C Cordell, Maikel Mansour, Austin R Pantel, Erin Schubert, Ali Nabavizadeh, Suyash Mohan, Eileen Maloney, Timothy Prior, Natalie Angeloni, Caroline Blessing, Suzanne Frangos, Leah Coghlan, E Paul Wileyto, Donald M O’Rourke, Steven Brem, Arati S Desai, Stephen J Bagley

**Affiliations:** Department of Medicine, Perelman School of Medicine at the University of Pennsylvania, Philadelphia, Pennsylvania, USA; Department of Neurosurgery, Perelman School of Medicine at the University of Pennsylvania, Philadelphia, Pennsylvania, USA; Department of Radiology, Perelman School of Medicine at the University of Pennsylvania, Philadelphia, Pennsylvania, USA; Department of Radiology, Perelman School of Medicine at the University of Pennsylvania, Philadelphia, Pennsylvania, USA; Department of Radiology, Perelman School of Medicine at the University of Pennsylvania, Philadelphia, Pennsylvania, USA; Department of Radiology, Perelman School of Medicine at the University of Pennsylvania, Philadelphia, Pennsylvania, USA; Department of Neurosurgery, Perelman School of Medicine at the University of Pennsylvania, Philadelphia, Pennsylvania, USA; Department of Neurosurgery, Perelman School of Medicine at the University of Pennsylvania, Philadelphia, Pennsylvania, USA; Department of Neurosurgery, Perelman School of Medicine at the University of Pennsylvania, Philadelphia, Pennsylvania, USA; Department of Neurosurgery, Perelman School of Medicine at the University of Pennsylvania, Philadelphia, Pennsylvania, USA; Department of Neurosurgery, Perelman School of Medicine at the University of Pennsylvania, Philadelphia, Pennsylvania, USA; Department of Neurosurgery, Perelman School of Medicine at the University of Pennsylvania, Philadelphia, Pennsylvania, USA; Department of Biostatistics, Epidemiology, and Informatics, Perelman School of Medicine at the University of Pennsylvania, Philadelphia, Pennsylvania, USA; Department of Neurosurgery, Perelman School of Medicine at the University of Pennsylvania, Philadelphia, Pennsylvania, USA; Department of Neurosurgery, Perelman School of Medicine at the University of Pennsylvania, Philadelphia, Pennsylvania, USA; Department of Medicine, Perelman School of Medicine at the University of Pennsylvania, Philadelphia, Pennsylvania, USA; Department of Medicine, Perelman School of Medicine at the University of Pennsylvania, Philadelphia, Pennsylvania, USA

**Keywords:** glioblastoma, IDH mutant glioma, niraparib, PARP inhibition, tumor treating fields

## Abstract

**Background:**

In preclinical models, tumor treating fields (TTFields) therapy promotes homologous repair deficiency (HRD), inducing potential sensitivity to poly (ADP-ribose) polymerase (PARP) inhibition. The aim of this study was to determine whether TTFields combined with the brain-penetrant PARP inhibitor niraparib has clinical efficacy in patients with recurrent high-grade glioma (HGG).

**Methods:**

We conducted a phase 2 trial of TTFields therapy concomitant with niraparib in patients with glioblastoma (*n* = 7) or IDH-mutant grade 4 astrocytoma (*n* = 2) that was recurrent after prior radiotherapy. Cohort A was a single-arm primary efficacy cohort with the Simon’s 2-stage design. Cohort B was a surgical window-of-opportunity cohort in which TTFields were administered for 5-7 days prior to surgery and then resumed post-operatively with niraparib. The primary endpoint was disease control rate in cohort A, defined as objective response or stable disease (SD) lasting at least 16 weeks.

**Results:**

The most common treatment-related adverse events were grade 1-2 dermatologic (scalp) toxicity in 67% of patients and grade 1-2 nausea in 67% of patients. In cohort A (*n* = 8), there were no objective responses, and 1 patient (12.5%) achieved SD lasting over 16 weeks. The efficacy benchmark to advance to Stage 2 was not achieved, and enrollment on Cohort A was terminated for futility. Cohort B (*n* = 1) was closed early due to slow accrual. In the single patient in Cohort B, TTFields-treated tumor tissue was negative for HRD.

**Conclusions:**

The combination of niraparib and TTFields therapy for recurrent HGG was safe and well tolerated but did not demonstrate an efficacy signal.

Key Points

- The study did not meet its primary efficacy endpoint.
- Combination treatment with TTFields and niraparib was safe and well tolerated.
- In the patient treated in the window-of-opportunity cohort, TTFields did not induce HRD in tumor tissue.

Importance of the Study

Recurrent high-grade gliomas are aggressive, incurable brain tumors with poor median overall survival. Based on preclinical data suggesting that TTFields therapy induces HRD in tumor cells, we performed a single-center phase 2 clinical trial to evaluate the efficacy of niraparib, a brain-penetrant PARP inhibitor, and TTFields in patients with recurrent grade 4 gliomas. Although this combination was overall safe and well tolerated, no signal of efficacy was detected. Further investigation is warranted to understand whether TTFields induces HRD in glioma cells in patients and to explore alternative means to sensitize gliomas to PARP inhibition.

Glioblastoma (GBM) is the most common malignant primary brain tumor in adults, comprising about 51% of all malignant central nervous system (CNS) tumors.^1^ GBM behaves aggressively with poor median overall survival (OS) of 12-15 months.^2^ Standard of care treatment includes maximal safe surgical resection followed by radiation and concomitant temozolomide (TMZ) chemotherapy, followed by maintenance TMZ and tumor-treating fields (TTFields) therapy.^3^^,^^4^ Despite this intensive treatment regimen, GBM universally recurs, and no therapeutic intervention has ever extended OS for patients with recurrent disease in a randomized clinical trial. Development of novel therapies for patients with recurrent diseases is urgently needed.

The clinical development of TTFields therapy represented a significant advance in first-line treatment for GBM, as it was the first intervention since TMZ to demonstrate a significant OS benefit.^5^ When administered with maintenance TMZ, the addition of TTFields further prolonged median OS to 20.9 months compared to 16 months with TMZ alone.^6^ In patients with recurrent GBM, TTFields as monotherapy resulted in similar OS compared to physician’s choice chemotherapy.^7^ The device, consisting of transducer arrays applied to the shaved scalp and used for at least 18 hours per day, applies low-intensity, intermediate-frequency (200 Hz) alternating electric fields to the brain.^6^ These fields interfere with cytokinesis and spindle formation in rapidly dividing cells, resulting in arrest of cell proliferation and cell destruction without impacting quiescent cells due to differences in the uniformity of electric fields in nondividing vs dividing cells.^8–10^ In addition to arresting cell division in vitro data suggests that TTFields impair homologous recombination, an important mechanism of double-stranded DNA break repair. In GBM cell lines, TTFields combined with radiation increased double-stranded DNA breaks compared to radiation alone, suggesting deficiency in the repair of these breaks.^11^ Furthermore, TTFields following radiation promoted the formation of RAD51 foci, which are sites of homologous repair.^12^ TTFields downregulated gene expression in the BRCA1 DNA-damage response pathway in non-small cell lung cancer and induced homologous repair deficiency (HRD) in ovarian cancer cells.^13–15^

HRD has therapeutic implications for certain tumors, often referred to as HRD-positive tumors, which have increased sensitivity to inhibitors of poly (ADP-ribose) polymerase (PARP), an enzyme responsible for the repair of single-stranded DNA breaks.^16^^,^^17^ Synthetic blockade of single-stranded DNA repair pathways via PARP inhibition combined with deficiencies in homologous repair can induce a “synthetic lethal” amount of genomic instability, resulting in apoptosis.^18^ PARP inhibitors have been shown to be effective in clinical trials treating ovarian and breast cancer in patients with homologous recombination defects due to germline BRCA1 or BRCA2 mutations.^19–21^ TTFields and PARP inhibitors have been demonstrated to be synergistic in preclinical studies across multiple cancer types. In glioma stem cells, TTFields induced increased replication stress when combined with a PARP inhibitor compared to TTFields alone.^22^ Furthermore, in a NSCLC cell line, TTFields and a PARP inhibitor followed by radiation synergistically induced cytotoxicity.^23^ Triple-negative breast cancer cell lines responded to combination therapy with a PARP inhibitor and TTFields by upregulating p21, an important G1-checkpoint protein that is lost in breast cancer cells.^24^ Similarly, TTFields and a PARP inhibitor synergistically increased apoptosis in ovarian cancer cells.^25^^,^^26^ These promising in vitro studies translated to mouse models of ovarian cancer that demonstrated prolonged survival following combination treatment with TTFields and a PARP inhibitor compared to TTFields alone.^27^

Based on these data, we hypothesized that TTFields may sensitize tumors to PARP inhibition in patients with high-grade glioma. We designed an open-label, single-center phase 2 clinical trial with a single-arm primary efficacy cohort and a window-of-opportunity cohort to evaluate the efficacy of niraparib and TTFields in patients with recurrent GBM or recurrent isocitrate dehydrogenase (IDH)-mutant grade 4 astrocytoma. Niraparib, a PARP inhibitor that is approved in the United States for ovarian cancer patients who had prior response to platinum-based chemotherapy regardless of germline BRCA mutations or HRD status, was chosen based on preclinical^28–30^ and human data suggesting adequate brain penetration to achieve desired pharmacodynamic effects.^31^^,^^32^

## Methods

### Patient Selection

Patients aged 22 years or older with a tissue-proven diagnosis of GBM or grade 4 IDH-mutant astrocytoma were recruited from the University of Pennsylvania. The latter group of patients were allowed given preclinical data suggesting that IDH-mutant tumors may be sensitive to PARP inhibition.^33^ Patients must have had recurrent disease following at least prior frontline radiotherapy, and any number of recurrences were allowed. Measurable contrast-enhancing disease was required, defined by at least 1cm x 1cm on magnetic resonance imaging (MRI) imaging within 28 days of starting study treatment. Key inclusion criteria included Karnofsky performance status (KPS) >= 60, live expectancy >3 months, adequate hematologic parameters, and adequate renal and hepatic function. Key exclusion criteria included treatment with TTFields in the last 6 months, prior treatment with a PARP inhibitor, patients with infratentorial or spinal tumor, and any known grade 3 or 4 anemia, neutropenia, or thrombocytopenia due to prior chemotherapy that persisted >4 weeks and was related to the most recent treatment.

### Study Design

**Figure 1.**
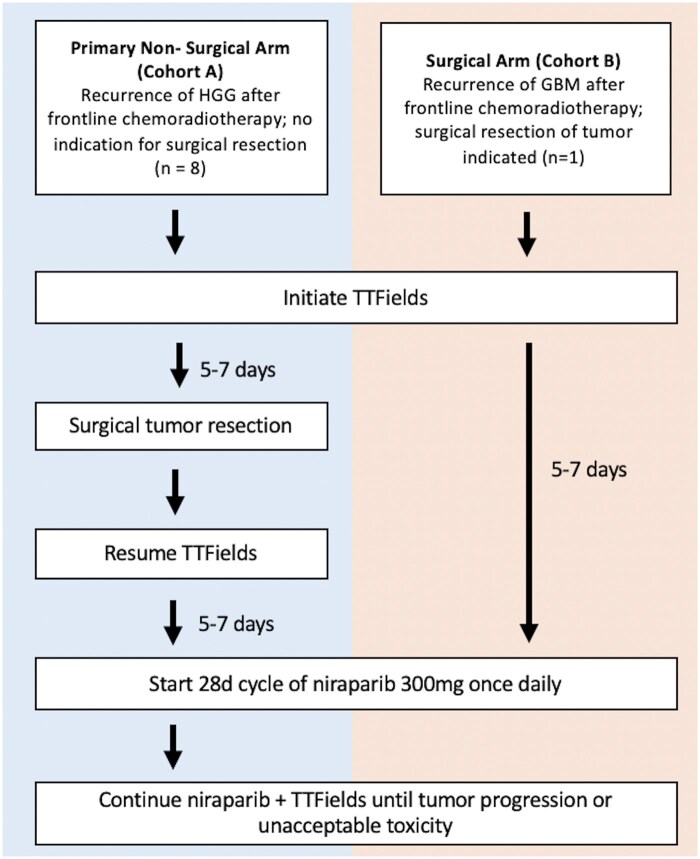
Study design. Abbreviations: GBM, glioblastoma; HGG, high-grade glioma; TTFields, tumor treating fields.

We conducted an open-label, single-center phase 2 clinical trial that consisted of 2 separate treatment cohorts (Figure 1). Cohort A enrolled patients who did not have a clinical indication for surgical resection of the recurrent tumor, while Cohort B enrolled patients with a clinical indication for surgical resection. The Simon’s 2-stage design was used for Cohort A, which was designed to assess the efficacy of the combination of TTFields and niraparib. A window-of-opportunity design was used for Cohort B, which was designed to test the hypothesis that TTFields therapy induces HRD in glioma tumor cells. In cohort A, subjects received TTFields with an encouraged usage rate of at least 18 hours per day followed by niraparib initiated 5 to 7 days after the start of TTFields. In cohort B, surgical tumor resection occurred 5 to 7 days after the start of TTFields, which was then resumed post-operatively following adequate healing of the craniotomy incision. Five to 7 days after resumption of TTFields, niraparib was initiated. Niraparib was dosed as 300 mg once daily on continuous 28-day cycles in both cohorts. Dose reductions were permitted for grade 3 or 4 toxicities, as determined by the investigator. Institutional Review Board (IRB) approval was obtained at the University of Pennsylvania, and the clinical trial was registered as NCT04221503.

### Endpoints

The primary endpoint was disease control rate (DCR) in Cohort A, defined as achievement of either objective response (OR), which included either complete response (CR) or partial response (PR), or stable disease (SD) per modified Response Assessment in Neuro-Oncology (mRANO) criteria lasting at least 16 weeks. Secondary endpoints were safety/tolerability, progression-free survival (PFS), and OS. Exploratory endpoints were pre- and post-TTFields [^18^F]FluorThanatrace (^18^F-FTT) positron emission tomography (PET) imaging for PARP-1 expression and assessment of homologous recombination deficiency (HRD) in TTFields-treated tumor tissue. For PET, patients were offered to consent for an optional exploratory analysis of ^18^F-FTT PET using the Ingenuity TF PET/CT machine (Philips Healthcare). This imaging technique quantifies PARP-1 expression, and a reduction in signal with PARP inhibitor treatment utilizing this ^18^F-FTT PET protocol has been shown in breast cancer to correlate with PARP inhibitor response.^34^  ^18^F-FTT PET imaging (54-90 minutes post 10.2-11.9 mCi) was obtained in 3 patients prior to the start of TTFields as a non-invasive measure of PARP-1 expression, with a single patient also imaged 6 days after TTFields initiation. For assessment of HRD in TTFields-treated tumor tissue, HRD status in TTFields-treated tissue obtained through surgical resection in patients enrolled in Cohort B was determined as positive or negative utilizing a test from Caris Life Sciences that identifies BRCA1/2 mutations and calculates a genomic scar score.^35^

### Assessments

Adverse events (AEs) were identified and graded using the National Cancer Institute Common Terminology Criteria for Adverse Events version 5.0 (CTCAE 5.0). AEs that could reasonably be attributed to the intervention based on temporality and investigator assessment were classified as treatment-related adverse events (TRAE). Hematologic parameters and renal and hepatic function were regularly assessed with serum laboratory testing, including at the time of screening and at least every 4 weeks. At the time of recruitment and every 8 weeks, patients underwent brain MRIs to monitor for tumor progression using mRANO criteria, which provides guidelines aimed to differentiate between progression and pseudoprogression due to radionecrosis.^36^

### Statistical Design and Analysis

The primary endpoint of DCR was analyzed by calculating the number of patients who achieved best response of SD, PR, or CR lasting at least 16 weeks and dividing that number by the total number of subjects enrolled. The primary (efficacy) hypothesis of this study was that the proportion of subjects who experienced disease control (i.e. achieve a confirmed best response to treatment of SD, PR, or CR lasting at least 16 weeks) would be 60% or higher. We tested this hypothesis against the null hypothesis of a DCR of 30% using the Simon’s 2-Stage Design for the primary study cohort (Cohort A). Stage 1 was able to enroll up to 8 evaluable subjects. If 4 or more subjects achieved disease control, then enrollment would be continued to Stage 2. In Stage 2, an additional 16 evaluable subjects would be enrolled, for a total of 24. If 11 or more subjects achieved disease control, then this regimen would be deemed worthy of further investigation. Overall, a sample size of 24 evaluable subjects was required in Cohort A for at least 80% power in the Simon’s 2-stage design. A 1-sided significance level was set at 0.05. The null hypothesis of 30% DCR was based on the historic DCR in subjects with recurrent GBM treated with standard of care lomustine across a number of contemporary clinical trials not involving bevacizumab.^37–39^ Secondary outcomes of median PFS and OS were calculated using the Kaplan-Meier method. Statistical analyses were performed with Stata version 14 (StataCorp). ChatGPT was utilized to generate Python code for the creation of survival curves.

## Results

### Baseline Characteristics

Nine patients were enrolled from December 2019 to March 2021 (Cohort A, *n* = 8; Cohort B, *n* = 1). Table 1 details the demographics and previous therapies of the trial participants. The median age of participants was 47, all of whom were male (Table 1).

**Table 1.** Patient characteristics (*N* = 9)

Patient characteristics	Value
Age at enrollment, years (median, IQR)	53 (47, 61)
Non-Hispanic, *n* (%)	9 (100)
Race, *n* (%)	
Asian	1 (11)
Black	1 (11)
White	7 (78)
Male gender, *n* (%)	9 (100)
KPS, *n* (%)	
90–100	2 (22)
70–80	7 (78)
IDH mutational status, *n* (%)	
IDH-wild type	7 (78)
IDH-mutant	2 (22)
Prior TMZ chemotherapy, *n* (%)	8 (89)
Prior radiotherapy dose, *n* (%)	
60 Gy	7 (78)
40 Gy	2 (22)
# prior relapses, *n* (%)	
1	3 (33)
2	6 (67)
Previously received bevacizumab, *n* (%)	3 (33)
Median time since original diagnosis, months (range)	9.6 (4.9, 30.3)

Abbreviations: IDH, isocitrate dehydrogenase; IQR, interquartile range; KPS, Karnofsky Performance Status; TMZ, temozolomide.

Among the 8 patients in cohort A, 6 had GBM and 2 had IDH-mutant grade 4 astrocytomas. Due to slow accrual during the COVID-19 pandemic, cohort B was terminated following recruitment of one patient, who had GBM. Across both cohorts, 3 patients (33%) were O6-Methylguanine-Methyltransferase (MGMT) promoter methylation positive. All patients previously received radiation, and 8 of 9 (89%) underwent prior treatment with TMZ. Three patients (33%) had received prior bevacizumab. No patients have ever received prior TTFields. Six patients (67%) had 2 prior recurrences, while 3 patients (33%) were in first recurrence at the time of enrollment.

### Safety and Toxicity

Overall, niraparib was well tolerated by all 9 patients across cohorts A and B. No patients requiring study drug discontinuation due to an AE. Table 2 displays the frequencies of TRAEs. The most common TRAE related to TTFields use was dermatologic toxicity, with 6 of 9 patients (67%) reporting dermatologic symptoms on the scalp (Table 2). Gastrointestinal TRAEs related to niraparib were also common with grade 1-2 nausea in 4 of 9 patients (44%), grade 1 vomiting in 4 of 9 patients (44%), grade 1 constipation in 2 of 9 patients (22%), and grade 2 anorexia in 2 of 9 patients (22%) (Table 2). Severe TRAEs were rare. One of 9 patients (11%) experienced grade 3 fatigue related to niraparib, and 1 of 9 patients (11%) developed grade 4 cerebral edema that was deemed possibly related to the combination of TTFields plus niraparib (Table 2). Dose reduction of niraparib to 200 mg was required in one patient due to grade 4 thrombocytopenia. No other patients require dose reduction.

**Table 2.** Treatment-related adverse events (*N* = 9)

Event	Any grade, No. (%)	Niraparib, No. (%)	TTFields, No. (%)	Grade ≥ 3, No. (%)	Niraparib, No. (%)	TTFields, No. (%)
**Blood and lymphatic system**	3 (33)	3 (33)		1 (11)	1 (11)	
Anemia	1 (11)	1 (11)				
Neutrophil count decreased	1 (11)	1 (11)				
Platelet count decreased	2 (22)	2 (22)		1 (11)	1 (11)	
**Cardiac**	**1 (11)**	**1 (11)**				
Sinus tachycardia	1 (11)	1 (11)				
**Gastrointestinal**	**6 (67)**	**6 (67)**	**2 (22)**			
Bloating	1 (11)	1 (11)				
Constipation	2 (22)	2 (22)				
Nausea	4 (44)	4 (44)	1 (11)			
Vomiting	4 (44)	4 (44)	2 (22)			
**General and admin site**	**2 (22)**	**2 (22)**		**1 (11)**	**1 (11)**	
Edema limbs	1 (11)	1 (11)				
Fatigue	1 (11)	1 (11)		1 (11)	1 (11)	
**Metabolism and nutrition**	**3 (33)**	**3 (33)**				
Anorexia	2 (22)	2 (22)				
Weight loss	1 (11)	1 (11)				
**MSK and connective tissue**	**1 (11)**	**1 (11)**				
Neck pain	1 (11)	1 (11)				
**Nervous system**	**4 (44)**	**3 (33)**	**4 (44)**	**2 (22)**	**1 (11)**	**2 (22)**
Ataxia	1 (11)		1 (11)	1 (11)		1 (11)
Cognitive disturbance	1 (11)	1 (11)	1 (11)			
Dysgeusia	1 (11)	1 (11)				
Edema cerebral	1 (11)	1 (11)	1 (11)	1 (11)	1 (11)	1 (11)
Headache	1 (11)	1 (11)	1 (11)			
Memory impairment	1 (11)	1 (11)	1 (11)			
Seizure	1 (11)		1 (11)			
**Renal and urinary**	**1 (11)**	**1 (11)**				
Creatinine increased	1 (11)	1 (11)				
**Skin and subcutaneous tissue**	**6 (67)**		**6 (67)**			
Erythema and/or pruritis	2 (22)		2 (22)			
Skin and subcutaneous tissue disorders—Other	1 (11)		1 (11)			
Skin infection, scalp	1 (11)		1 (11)			
Skin ulceration	2 (22)		2 (22)			
Small area of irritation on scalp	1 (11)		1 (11)			

Abbreviations: admin, administration; MSK, musculoskeletal; TTFields, tumor treating fields. Bolded rows represent categories of adverse events by system organ class.

### Efficacy

**Figure 2.**
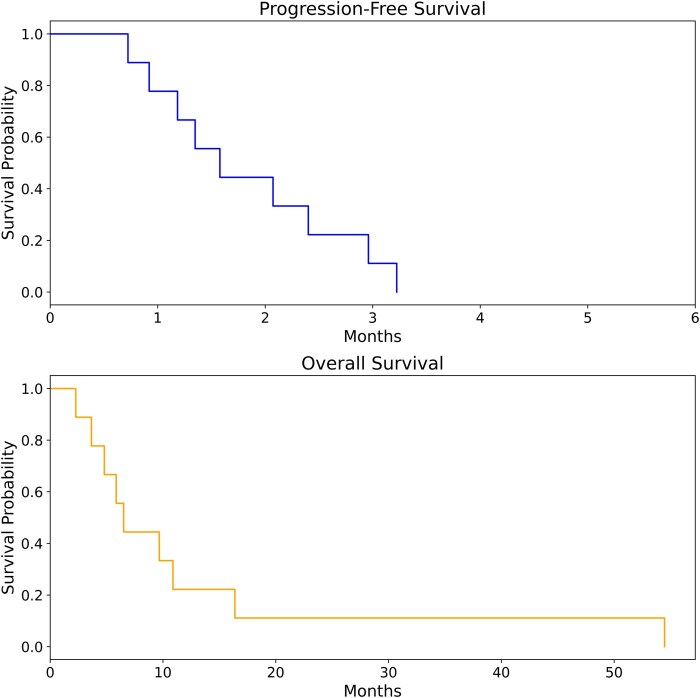
Progression-free survival (PFS) and overall survival (OS) in months. Median PFS = 1.3 months (95% CI 0.7-3.0 months). Median OS = 6.5 months (95% CI, 2.3-16.4 months).

Across all 9 patients, TTFields usage was adequate, with 80.7% (19.4 hours/day) average utilization at the time of niraparib initiation. No ORs were observed. The efficacy benchmark to advance to Stage 2 was therefore not achieved, and enrollment in Cohort A was terminated for futility following completion of Stage 1. In Cohort A, median PFS was 1.3 months (95% CI 0.7-3.0 months), and median OS was 6.5 months (95% CI 2.3—16.4 months) (Figure 2). One of 8 patients (12.5%) in Cohort A, 47-year-old with an IDH wild type, and MGMT unmethylated tumor in the first recurrence, achieved SD lasting at least 16 weeks. This patient had a craniotomy for tumor debulking approximately 4 weeks prior to study enrollment, with pathology confirming residual/recurrent GBM, and the screening MRI at the time of trial enrollment showed a 4.4 x 2.2 cm mass in the left parietotemporal region with elevated perfusion and permeability metrics.

**Figure 3.**
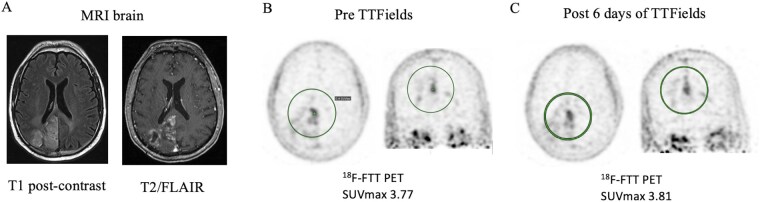
(A) MRI brain of cohort B patient pre-TTFields (tumor treating fields). T1 post-contrast image (left) and T2/FLAIR image (right). (B) [^18^F]FluorThanatrace (^18^F-FTT) positron emission tomography (PET) imaging for PARP-1 expression of Cohort B patient pre-TTFields. SUV_max_ = 3.77. (C) ^18^F-FTT PET imaging 6 days post TTFields demonstrating no difference in PARP-1 expression. SUV_max_ = 3.81.

In the 3 patients who underwent ^18^F-FTT PET prior to the start of TTfields, ^18^F-FTT uptake was seen in all lesions above background, suggesting PARP-1 expression in these tumors. No change in ^18^F-FTT was seen in the single patient, a 53-year-old male with non-MGMT methylated GBM, imaged following initiation of TTFields (SUVmax 3.77 pre-TTFields and 3.81 post-TTFields), suggesting that PARP-1 expression remained unchanged in the tumor (Figure 3). In Cohort B, the single patient treated had a PFS of 2.1 months and OS of 5.9 months. HRD testing performed on post-TTFields tumor tissue from this patient was negative.

## Discussion

In this open-label, single-center phase 2 clinical trial, combination therapy with niraparib and TTFields in patients with recurrent grade 4 glioma was feasible, safe, and well tolerated. However, the study did not meet the prespecified endpoint for efficacy. While reasons for the lack of efficacy signal are uncertain, it is unlikely that limited penetration of niraparib into the tumor tissue was a major contributor. In a phase 0/2 trigger trial of niraparib administered prior to resection of newly diagnosed GBM, all patients (*n* = 46) demonstrated pharmacologically relevant concentrations and measurable pharmacodynamic effects of niraparib in tumor tissue.^32^ Instead, TTFields may not have induced sufficient levels of HRD in vivo to adequately sensitize the tumor to PARP inhibition. It is also possible that TTFields induced HRD, but that this alone was inadequate to result in glioma cell death due to other resistance mechanisms, such as the presence of alternative DNA damage response pathways. Our study was limited in its ability to assess HRD status due to limited accrual of the surgical arm, with only one patient being enrolled. However, it is notable that this patient’s tumor was HRD negative after exposure to TTFields. Similarly, PARP activity, as measured by ^18^F-FTT PET, was not induced by 7 days of TTFields treatment in a single patient with pre- and post-TTFields ^18^F-FTT PET.

PARP inhibition to treat newly diagnosed and recurrent GBM has been evaluated in prior trials with mixed results. In a phase 0/2 trigger trial of niraparib in patients with newly diagnosed GBM, patients were treated with niraparib prior to planned resection.^32^ Following ex vivo radiation after resection, 73% of patients (24/33) exhibited PARP inhibition. Patients that demonstrated PARP suppression with niraparib and had MGMT unmethylated tumors were invited to advance to phase 2, where they received concomitant niraparib and radiotherapy followed by maintenance niraparib. Although mature OS data is lacking, median PFS at time of publication was 11.7 months,^32^ which is higher than the historical 6.9-month PFS observed in patients undergoing standard chemoradiotherapy.^4^ These promising data highlight the possible synergy of PARP inhibition and radiation in patients with newly diagnosed GBM as well as the utility of selecting responders to niraparib through ex vivo studies. In the recurrent setting, however, the clinical data for PARP inhibition has been less encouraging. In a trial for recurrent GBM utilizing another PARP inhibitor, olaparib, in combination with chemotherapy, only 14 of 36 (39%) patients were progression-free at 6 months, which was determined by the authors to be insufficient to warrant future investigation.^41^ Another phase 2 study in the recurrent setting tested talazoparib with carboplatin for patients with genomic enrichment in the DNA damage repair pathway and demonstrated a median PFS of only 3.5 months.^42^ Whether patients with IDH-mutant tumors may respond better to PARP inhibition, which has been suggested by preclinical data,^33^ is an active area of investigation with a recently completed clinical trial in adults^43^ and an ongoing clinical trial in adolescents and young adults (NCT03749187). However, a multicenter phase 2 trial of olaparib in IDH-mutant astrocytoma did not meet its prespecified efficacy endpoint.^44^ Novel combinations with PARP inhibitors are also being actively explored, including combination PARP and VEGF inhibition (NCT02974621).

Overall, this study of PARP inhibition with niraparib in combination with TTFields did not provide an efficacy signal in patients with recurrent high-grade glioma. However, given the abundance of preclinical studies consistently supporting the biological rationale for this combination, future studies could consider exploring PARP inhibition and TTFields as maintenance therapy in newly diagnosed GBM or in other solid tumors where TTFields has already displayed efficacy, such as pancreatic cancer^45^ and non-small cell lung cancer.^46^ In addition, based on preclinical data suggesting that HRD and PARP sensitivity were most apparent when combining TTFields and ionizing radiation,^11^^,^^12^ future studies may consider combining TTFields with ionizing radiation and PARP inhibition to induce greater clinical efficacy.

## Data Availability

All data analyzed will be made available upon reasonable request.
